# A Case Study of a Rare Undifferentiated Spindle Cell Sarcoma of the Penis: Establishment and Characterization of Patient-Derived Models

**DOI:** 10.3390/genes15040424

**Published:** 2024-03-28

**Authors:** Ariane Cavalcante dos Santos Sousa, Bruno Leonardo Nascimento Correa Fernandes, Jeronimo Paulo Assis da Silva, Paulo Roberto Stevanato Filho, Luiza Bitencourt de Carvalho Terci Coimbra, Adriano de Oliveira Beserra, Ana Luiza Alvarenga, Giovanna Maida, Camila Tokumoto Guimaraes, Ingrid Martinez Nakamuta, Fabio Albuquerque Marchi, Camila Alves, Martina Lichtenfels, Caroline Brunetto de Farias, Bruna Elisa Catin Kupper, Felipe D’Almeida Costa, Celso Abdon Lopes de Mello, Dirce Maria Carraro, Giovana Tardin Torrezan, Ademar Lopes, Tiago Goss dos Santos

**Affiliations:** 1 Clinical and Functional Genomics Group, A.C. Camargo Cancer Center, Sao Paulo 01508-010, Brazil; ariane.sousa@accamargo.org.br (A.C.d.S.S.); luizabctc@gmail.com (L.B.d.C.T.C.); adriano.beserra@accamargo.com.br (A.d.O.B.); ana.alvarenga@accamargo.org.br (A.L.A.); g.maida@accamargo.org.br (G.M.); camila.tokumoto@accamargo.org.br (C.T.G.); dirce.carraro@accamargo.org.br (D.M.C.); giovana.torrezan@accamargo.org.br (G.T.T.); 2 National Institute of Science and Technology in Oncogenomics and Therapeutic Innovation, Sao Paulo 01508-010, Brazil; 3 Graduate Program of A.C.Camargo Cancer Center, Sao Paulo 01508-020, Brazil; ingrid.nakamuta@gmail.com; 4 Sao Lucas Hospital Rede D’Or, Sergipe Emergency Hospital Oncology Service, Aracaju 49081-060, Brazil; brunoleofernandes@hotmail.com; 5 Real Institute of Oncological Surgery, Real Hospital Português, Recife 52010-075, Brazil; jeronimoassis@gmail.com; 6 Reference Center in Sarcoma, A.C. Camargo Cancer Center, Sao Paulo 01509-900, Brazil; paulo.stevanato@accamargo.org.br (P.R.S.F.); bruna.catin@accamargo.org.br (B.E.C.K.); felipe.costa@accamargo.org.br (F.D.C.); ademar.lopes@accamargo.org.br (A.L.); 7 Heart Institute of School of Medicine, University of Sao Paulo, Sao Paulo 05403-900, Brazil; 8 Center for Translational Research in Oncology, Cancer Institute of the State of Sao Paulo (ICESP), Clinical Hospital of the University of Sao Paulo Medical School (HCFMUSP), Sao Paulo 01246-000, Brazil; fabio.marchi@hc.fm.usp.br; 9 Ziel Biosciences, Department of Translational Research, Porto Alegre 90050-170, Brazil; eng.camilaalves@gmail.com (C.A.); martinalichtenfels@hotmail.com (M.L.); carolbfarias@gmail.com (C.B.d.F.); 10 Anatomic Pathology Department, A.C. Camargo Cancer Center, Sao Paulo 01509-900, Brazil

**Keywords:** sarcomas, preclinical models, functional precision oncology, chemosensitivity

## Abstract

Rare sarcomas present significant treatment challenges compared to more prevalent soft tissue sarcomas due to limited treatment options and a poor understanding of their biology. This study investigates a unique case of penile sarcoma, providing a comprehensive morphological and molecular analysis. Through the creation of experimental patient-derived models—including patient-derived xenograft (PDX), 3D, and monolayer primary cultures—we successfully replicated crucial molecular traits observed in the patient’s tumor, such as smooth muscle actin and CD99 expression, along with specific mutations in genes like *TSC2* and *FGFR4*. These models are helpful in assessing the potential for an in-depth exploration of this tumor’s biology. This comprehensive approach holds promise in identifying potential therapeutic avenues for managing this exceedingly rare soft tissue sarcoma.

## 1. Introduction

Soft tissue sarcomas (STSs) represent a group of rare neoplasms characterized by diverse morphological patterns of mesenchymal cells [1,2]. STSs account for roughly 1% of malignant tumors in adults and 7% in children [3], with an annual incidence of approximately five to six new cases for every 100,000 individuals and can emerge at any age and in nearly any anatomical site [1,4]. High-grade STSs are known for aggressive behavior, and approximately 50% of patients with high-risk tumors will develop distant metastasis, mainly to the lungs [1].

Due to their mesenchymal origin, STSs constitute a very heterogeneous group of tumors, comprising over 100 subtypes [5]. This substantial diversity often results in distinct clinical behaviors and imposes tailored therapeutic strategies [6]. Surgery is the main treatment for non-metastatic disease, and chemotherapy and radiation therapy can improve the outcomes for a specific group of patients [7,8].

Patients diagnosed with rare sarcomas face an even more challenging scenario. Ultra-rare sarcomas are defined by an incidence rate of approximately ≤1 per 1,000,000 individuals. These entities are so exceptionally infrequent that conducting prospective clinical trials becomes notably difficult [9]. Therefore, there is a clinical need to gather molecular data on ultra-rare sarcomas and create experimental models to explore the specific vulnerabilities of these tumors. In this report, we present a comprehensive and genomic molecular characterization of a unique case of a rare presentation of a high-grade undifferentiated spindle cell sarcoma affecting the penis with comprehensive molecular characterization. Additionally, to further explore the tumor’s biological characteristics further, we successfully established both a patient-derived xenograft model and a tumorigenic cell line.

## 2. Case Presentation

**Figure 1:**
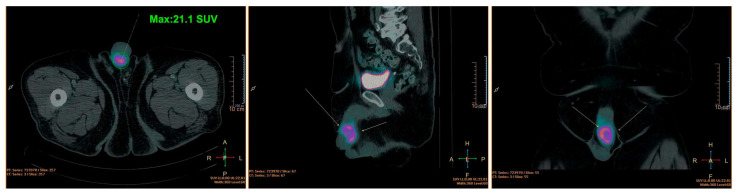
PET-CT showing the presence of an anomalous concentration area due to the lesion in the penile body. The colors indicate an anomalous concentration area of 18F-DG and correspond to a site of abnormal local cellular metabolic activity (active lesion).

A 56-year-old male patient presented with a progressively growing lesion at the base of the penis. He has a medical history of diabetes and hypertension and a family history of lung cancer. On physical examination, a tumoral mass was identified within the penile body, originating at the junction of the glans and the body, extending to approximately 2 cm towards the pubic region (Figure 1). The patient underwent true-cut biopsy and the diagnosis was high-grade spindle cell sarcoma. Imaging tests revealed a heterogeneous mass in the middle third of the penis, adjacent to the right corpus cavernosum. This mass appeared predominantly hypoechoic and exhibited intense vascularization in color Doppler imaging, measuring 4.0 cm × 3.0 cm. Subsequent pelvic magnetic resonance imaging (MRI) revealed an expansive lesion with irregular, poorly defined contours located in the transition between the glans and the cavernous bodies. This lesion exhibited slightly heterogeneous contrast enhancement with pronounced hypo signal foci observed in the apparent diffusion coefficient (ADC) images, indicating high cellularity. The dimensions measured were 4.1 cm × 3.9 cm × 3.2 cm. In addition, a computed tomography scan of the pelvis revealed the presence of a heterogeneous expansive lesion located at the transition between the glans and cavernous bodies, measuring approximately 4.1 cm, which was consistent with a primary tumor site. The exam also indicated the presence of a small calcified nodule in the right upper lobe, measuring 0.3 cm. The liver appeared with normal dimensions, with a hypodense nodular image in the periphery of the hepatic segment V, measuring 1.3 cm of indeterminate appearance. A PET-CT scan further confirmed the presence of an abnormal 18-FDG concentration in the pelvic region, corresponding to a lesion in the penile body with a standardized uptake value (SUV) of 21.1. No other regions of abnormal 18-FDG concentration were identified in the other anatomical areas examined, confirming the absence of distant metastasis (Figure 1).

The patient underwent a total penectomy and recovered uneventfully. The pathologic report showed a 4.5 cm lesion characterized by congested vessels, the presence of necrosis and mitosis, and foci of perineural invasion. Immunohistochemistry showed staining only for smooth muscle actin (SMA) and vimentin. The final diagnosis was a high-grade undifferentiated spindle cell sarcoma, located in the spongy and cavernous body of the penis.

After a multidisciplinary tumor board discussion, no adjuvant therapy was recommended. Eleven months after surgery, the patient presented distant metastasis (lung and bone) and initiated palliative chemotherapy with doxorubicin. After four cycles, the patient presented with disease progression. The patient received gemcitabine + docetaxel as the second-line regimen and pazopanib in the third line and died due to the progression of disease 10 months after the diagnosis of cancer relapse.

## 3. Methods

### 3.1. Animals

Female and male 2–4-month-old NSG mice were obtained from the Jackson Laboratory (Bar Harbor, ME, USA). NSG colonies were maintained in the A.C. Camargo animal facility following the National Institutes of Health (USA) and institutional guidelines for animal welfare and experimental conduct.

### 3.2. Patient-Derived Xenograft (PDX)

Fresh tumor fragments obtained from a surgical specimen were minced into minor fragments measuring between 1 and 3 mm and inoculated into the dorsal subcutaneous region in NSG animals that were previously anesthetized, using 11 G cancer implant needles (Cadence Science, Plainfield Pike, Cranston, RI 02921, USA). The excess material was cryopreserved by vitrification in liquid nitrogen. A maximum of five fragments were implanted into each animal, and tumor growth was monitored. Tumorigenesis tests were also performed with primary PDX culture. A total of 1 × 10^6^ cells were inoculated into the right flank of NSG mice.

### 3.3. Monolayer and 3D Cell Cultures

Fresh and cryopreserved tumor fragments were used to establish primary cell cultures. The tumor mass was minced using a scalpel followed by hydromechanical dissociation. The homogenate was subjected to enzymatic digestion in a collagenase and trypsin mixture (1:1) for 30 min to 2 h at 37 °C under agitation. After enzyme washout, cells were filtered in a 100 µm cell strainer, plated in 75 cm^2^ bottles, and kept in an incubator at 37 °C and 5% CO_2_. Cell stocks were cryopreserved and maintained in liquid nitrogen.

Three-dimensional culture was performed using magnetic levitation [10]. Tumor fragments were dissociated with Collagenase I, and cells were incubated with poly-L-lysine and iron oxide nanoparticles (10 µL every 1000 cells) (n3D Biosciences, Houston, TX 77030, USA) for 1 h in an orbital shaker. Cells were resuspended in culture medium (DMEM High + 20% FBS) to remove excess nanoparticles. The cells were plated in cell-repellent microplates in an oven at 37 °C and 5% CO_2_ under concentration magnets for 48 h. After this period, the concentration magnets were replaced by a levitation plate. After 1 week, the organoids generated by this protocol were fixed using 4% paraformaldehyde and embedded in paraffin to perform immunohistochemistry.

### 3.4. Chemoresistance Platform

Primary cells were maintained in T-75 flasks and when they reached confluence, 5 × 10^3^ cells were tested on the chemoresistance platform (Bioverso^®^, Ziel Biosciences, Porto Alegre, RS 90050-170, Brazil). The platform consisted of a 96-well plate containing single chemotherapeutic agents in different concentrations. The QT agents were doxorubicin (1 and 5 µM), gemcitabine (0.1, 1, and 10 µM), cyclophosphamide (2 and 4 mM), etoposide (50 and 100 µM), docetaxel (0.5 and 1 µM), paclitaxel (0.1 and 1 µM), and vincristine (1 and 5 µM). Cells were incubated for 72 h and cell viability was assessed using an MTT assay (5 mg/mL in PBS). Briefly, cells were incubated with MTT solution for 3 h. The medium was removed, isopropanol was added to each well, and absorbance was measured on a BioRAD—iMark MicroplateTM Reader (Bio-Rad, Shinagawa, Tokyo).

### 3.5. Immunohistochemistry and Immunofluorescence

Immunohistochemical staining was performed on the automated Ventana Discovery XT system (Ventana Medical Systems, Tucson, AZ, USA) using commercially available antibodies against smooth muscle actin (clone 1A4, Dako), cytokeratin (clone AE1-AE3, Dako), CD34 (ClassII, clone QBEnd 10, Dako), Desmin (clone D33, Dako), Myogenin (clone F5D, Dako), CD99 (MIC 2 gene product Ewing’s Sarcoma Marker/clone 12E7, Dako), and S-100 (polyclonal rabbit, Dako). Images were acquired using an Aperio Imagescope (Leica Biosystems, Nußloch, Heidelberger Str. 17-19, Germany). The cultured cells were immunofluorescent fixed with 4% paraformaldehyde for 20 min, washed twice with PBS, and incubated overnight with primary antibodies, followed by incubation with fluorescence-conjugated AlexaFluor 488 antibodies. Images of cultured cells were obtained using a Leica SP5 TCS SP II confocal microscope.

### 3.6. Exome Sequencing and Analysis

DNA extraction from the patient’s tumor, leukocytes, and PDX tissue was performed using Phenol/Chloroform/Isoamyl Alcohol, 25:24:1 (*v*/*v*). Whole-exome sequencing was performed with Agilent SureSelect XT library on an Illumina platform, aiming for a mean 100× coverage. Genomic DNA underwent prior quality control (QC) steps, followed by library preparation and sequencing. The generated data were analyzed by the Computational Biology and Biomarkers Laboratory team using the Genome-Analysis-Toolkit (GATK) pipeline for somatic variant analysis. Alignment of FASTQ files with the human reference genome (hg19/GRCh37 version) was performed using BWA software (version 0.7.17-r1188) in MEM command. BAM files proceeded to the pre-processing stage using GATK software (version 3.8) with MarkDuplicates, BaseRecalibrator, and PrintReads steps with default parameters. Sequencing data from tumors generated in PDX models tend to contain reads related to the murine host. Data processing utilized the XenofilteR package [11] for the exclusion of murine reads, generating a new BAM file corresponding to sequences that aligned with the reference genome. Subsequently, the data were analyzed following the same aforementioned steps, following GATK best practices.

Identified variants underwent further annotation and prioritization with VarSeq™ software (Golden Helix^®^ version 2.5.0), where filters were applied to further refine the data, leaving only high-quality variants. Variants were selected when present in >10 reads (at least 3 reads in each strand) and with allele frequency >5%. To identify variants more likely to be protein damaging, we then selected only the coding variants leading to loss of protein function (LoF variants) or missense variants that had a REVEL score above 0.5.

## 4. Results

**Figure 2:**
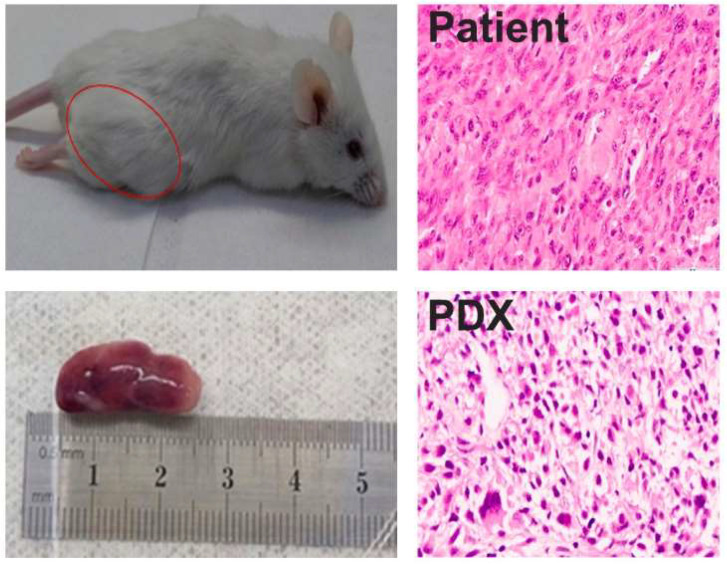
Generation of PDX model of ultra-rare penile sarcoma. (**Left**) Representative images from NSG mouse with ectopic tumor. (**Right**) HE staining from patient’s tumor and PDX showing similar morphologic patterns.

Considering the rarity of this tumor type, we generated a patient-derived xenograft (PDX) to establish an experimental model for this unique tumor. Surgical fragments were inoculated via the ectopic (subcutaneous) route. Two months later, we successfully identified the tumor mass, which was subsequently resected and serially passaged to establish the tumorigenic model. In Figure 2, representative images are presented illustrating the Hematoxylin and Eosin (HE) staining of both the patient’s tumor and the PDX.

**Figure 3:**
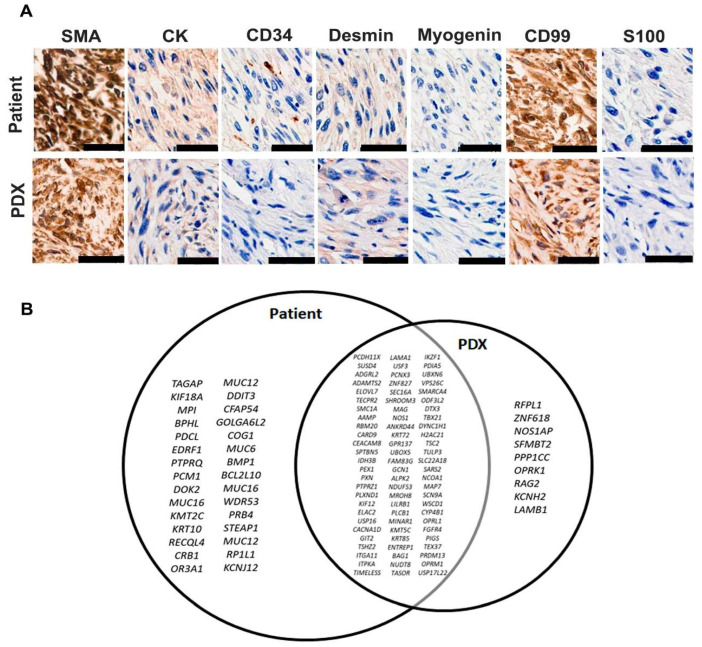
Comparison of penile sarcoma from patient with PDX models. (**A**) Immunohistochemical evaluation with major sarcoma markers, showing positivity for SMA and CD-99 and negative labeling for cytokeratin, CD-34, Desmin, Myogenin, and S-100. Calibration bars = 50 µm). (**B**) Venn diagram showing shared and exclusive mutations identified in patient’s tumor and PDX.

Subsequently, we conducted a comprehensive characterization of the PDX using immunohistochemistry and exome sequencing. Figure 3A shows the immunohistochemical comparison between the patient’s original tumor and the PDX model while preserving the tumor characteristics, as indicated by markers such as SMA, Desmin, CD99, CD44, Myogenin, S-100, and cytokeratin (CK). The presence of positive staining for SMA, CD99, CK, and Desmin underscores the striking similarity between these specimens. Supplementary Table S1 presents a comprehensive list of mutated genes, while Figure 3B illustrates the shared and exclusive mutations identified in the patient’s tumor and the PDX through exome sequencing. Although none of the identified alterations are classified as driver mutations in the consulted databases (cBioPortal and OncoKb), they have the potential to damage protein function. Notably, certain alterations, such as *TSC2*, *FGFR4*, and *SMARCA4*, present opportunities for pharmacological targeting using specific drugs, a prospect to be thoroughly investigated in forthcoming studies. A detailed list of the altered genes is presented in Supplementary Table S1. Concerning the variant allele frequency (VAF), our analysis indicates a similar percentage in both the patient’s tumor and the PDX, reinforcing the fidelity of the PDX model in mirroring the major molecular alterations observed in the original tumor (Supplementary Table S1).

Unfortunately, we were not able to evaluate the presence of fusions in the patient’s tumor, since analysis using Archer FUSIONPlex Sarcoma v1 panel was inconclusive.

**Figure 4:**
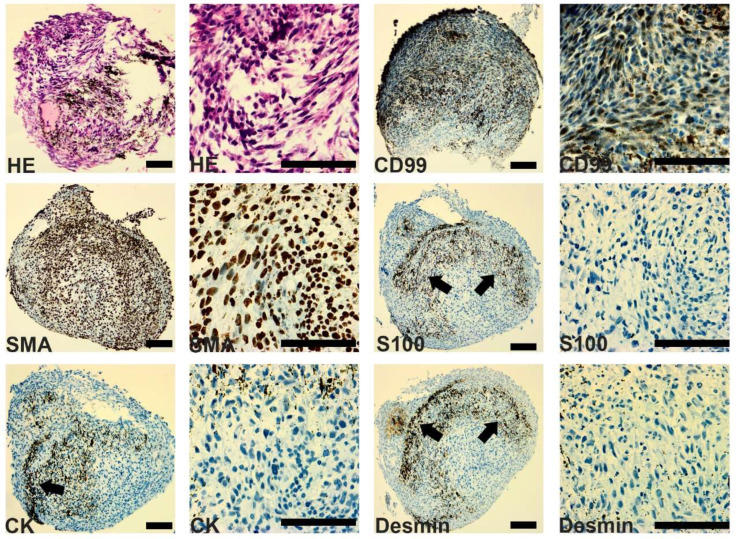
Patient-derived organoids generated using magnetic levitation. After fixation and paraffin embedding, the organoids were sectioned (3 μm) and the slides were stained with Hematoxylin–Eosin (HE). Immunohistochemistry performed with proteins commonly expressed in PMS (CD99, SMA, CK, S100, and Desmin). The arrow indicates non-specific staining that originated from nanoparticles. Images obtained by Aperio. Calibration bar = 100 μm.

Furthermore, we generated patient-derived organoids (PDOs) using the magnetic levitation approach [12]. It was possible to verify that the PDOs expressed proteins such as CD99 and SMA and did not express CK, S100, and Desmin (Figure 4).

**Figure 5:**
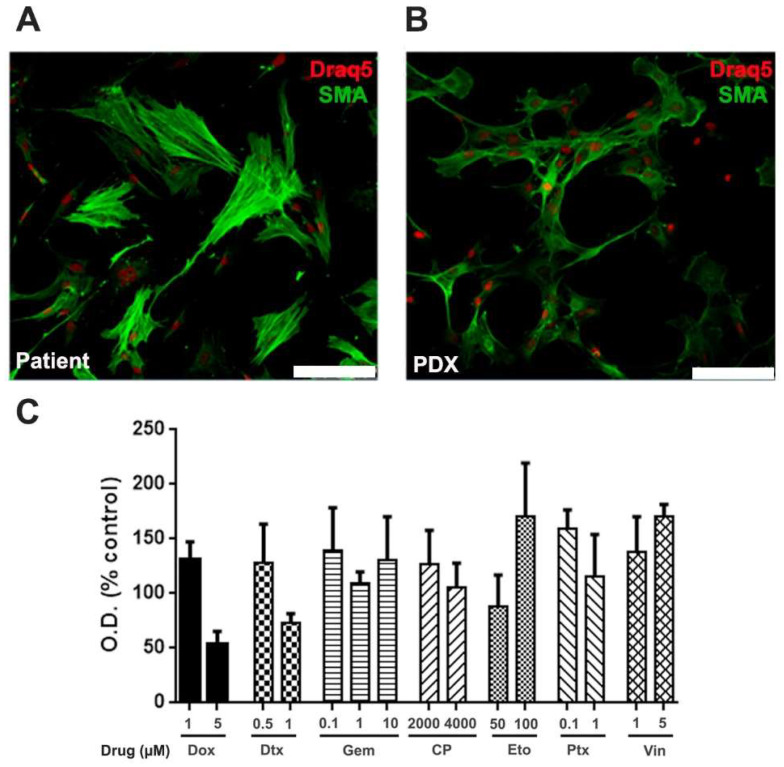
Generation of primary cultures from patient’s tumor. Upper panels represent primary cells derived from the patient’s tumor (**A**) or PDX (**B**). Cells are labeled with SMA (green) and Draq5 (red), calibration bar = 100 μm. (**C**) Cells were incubated in the presence of doxorubicin, gemcitabine, cyclophosphamide, etoposide, docetaxel, paclitaxel, and vincristine. After 72 h, cell viability was assessed by MTT.

Finally, we established both monolayer and 3D cell culture lineages from this specimen, enhancing our experimental model platform. Cells were immortalized through sequential passaging, and their tumorigenicity capacity was confirmed when inoculated into NSG mice, while preserving the tumor characteristics, as indicated by monolayer culture and markers of immunofluorescence analysis such as MDM2, SMA, Desmin, CD99, CD44, Myogenin, S-100, and CK, as depicted in Figure 5A,B.

Primary cells were exposed to various standard-of-care sarcoma chemotherapeutic drugs in a chemoresistance platform (Bioverso, Ziel Biosciences). The cells demonstrated consistent chemoresistance across most treatments, with only low resistance observed at the highest concentration of doxorubicin (5 µM) and docetaxel (1 µM) after 72 h of treatment (Figure 4C). These findings mirror the outcomes observed in the patient who presented disease progression following first-line treatment with doxorubicin and subsequent second-line therapy with gemcitabine plus docetaxel.

## 5. Discussion

In this study, we explored a rare case of penile sarcoma through the development of both in vivo and in vitro models to uncover specific molecular and genomic traits of this tumor. Our molecular analyses unveiled significant alterations within the patient’s tumor that were also found in the PDX model.

Malignant penile tumors are exceedingly rare, with an incidence ranging from 0.5 to 5 cases per 100,000 men [13]. Notably, primary penile sarcoma constitutes a minor fraction, less than 5% of all penile malignancies [13]. Histological subtypes encompass various rare sarcomas, including epithelioid sarcoma, Kaposi’s sarcoma, angiosarcoma, leiomyosarcoma, rhabdomyosarcoma, osteosarcoma, Ewing’s sarcoma, clear cell sarcoma, and spindle cell sarcoma [14]. Among these, spindle cell sarcoma stands out as an exceptionally uncommon penile tumor, setting it as an ultra-rare sarcoma [9].

Given the rarity of these ultra-rare tumors, the integration of preclinical studies assumes paramount importance in advancing novel therapies. Such studies enable a comprehensive exploration of unique molecular targets, gene functions, and biomarkers, significantly enhancing our understanding and the development of effective treatments [15].

In our study, we identified somatic mutations that were present both in the primary tumor and in the PDX, and some of these mutations were involved in relevant tumorigenic pathways. However, the use of next-generation sequencing, as well as specially acquired somatic mutations, to search for molecular targets in sarcomas seems to benefit a very small group of patients, especially when limited to detecting only point mutations. One of the largest series reporting molecular alterations in sarcomas used the Foundation Medicine platform and identified that approximately one-third of the patients presented any somatic mutation, but only a minority benefited from a target-driven therapeutic approach [16].

Among the pivotal preclinical models explored, PDXs for cancer research play a crucial role. These models largely retain the key histological and genetic characteristics of the patient’s tumor across passages [17], offering insights into the individuality and heterogeneity of tumors, including the unpredictability of tumor progression [18]. Proven to be predictive of clinical outcomes, PDX models are instrumental in preclinical drug evaluations and the identification of new biomarkers [19,20,21].

Additionally, our study delved into patient-derived 3D models (tumor organoids), opting to utilize tumor samples derived from a PDX due to their capacity for in vivo tumor growth. Patient-derived organoids (PDOs), characterized as 3D cultures derived from primary cells, maintain both architectural and functional similarity with their originating organs. This model preserves the genetic characteristics present in the original tumors [22]. The applications of organoids in precision medicine are multifaceted. They enable deeper insights into molecular alterations, the identification of potential biomarkers, and expedited drug sensitivity testing compared to the use of PDX models [22,23].

Finally, functional precision oncology represents an innovative treatment approach wherein cytotoxic drugs are tested against patient-derived cancer cells, harnessing the unique characteristics of individual tumors, maximizing cytotoxic therapy, identifying tumor resistance, and minimizing adverse effects [24]. In our study, the fidelity of patient-derived cells in mirroring the chemoresistance profile observed in the patient underscores the platform’s ability to predict drug resistance patterns with remarkable precision.

In the same way that the characterization of a rare sarcoma brings valuable insights, it also presents a limitation as the results cannot be generalized, impacting the applicability of the findings to a broader population beyond undifferentiated spindle cell sarcomas.

Taken together, generating experimental models to test therapies not only aids in predicting outcomes in patients but also unveils nuances that could significantly enhance diagnostic capabilities, particularly in biomarker identification, and improve treatment strategies.

## Data Availability

Data is available on request due to restrictions.

## Supplementary Materials

The following supporting information can be downloaded at: https://www.mdpi.com/article/10.3390/genes15040424/s1, Table S1: Mutation profile in patient’s tumor and PDX.
