# Supplementary material for: A Case Study of a Rare Undifferentiated Spindle Cell Sarcoma of the Penis: Establishment and Characterization of Patient-Derived Models
Source: Genes (Basel). 2024 Mar 28;15(4):424. doi: 10.3390/genes15040424 (PMC11049969; doi:10.3390/genes15040424)
Supplement: Supplementary file 1 [file genes-15-00424-s001.zip › genes-2929711-supplementary.pdf]

Supplementary Table S1 – Mutation profile in patient's tumor and PDX

					Patient's tumor	PDX
	**Gene**	**Effect**	**Variant codon**	**Variant protein**	**VAF (%)**	**VAF (%)**
Shared Alterarions	*PCDH11X*	Missense	c.112G>T	p.Val38Phe	100.00	100.00
	SUSD4	Missense	c.838C>G	p.Leu280Val	89.53	61.36
	ADGRL2	Missense	c.4000G>A	p.Glu1334Lys	87.39	79.69
	ADAMTS2	LoF	c.3544C>T	p.Arg1182Ter	81.25	75.81
	ELOVL7	Missense	c.512C>T	p.Thr171Ile	76.97	68.70
	TECPR2	Missense	c.1235C>T	p.Ser412Leu	69.23	64.71
	SMC1A	Missense	c.3119A>G	p.Glu1040Gly	67.42	43.62
	AAMP	LoF	c.122-1G>A	n.a.	62.96	67.50
	RBM20	Missense	c.419C>T	p.Pro140Leu	62.01	63.09
	CARD9	Missense	c.1399G>T	p.Ala467Ser	60.64	63.27
	CEACAM8	Missense	c.215C>G	p.Thr72Arg	59.18	52.21
	SPTBN5	Missense	c.1984G>A	p.Val662Met	57.24	51.97
	IDH3B	Missense	c.395T>C	p.Leu132Pro	55.65	48.62
	PEX1	Missense	c.548G>A	p.Arg183Gln	55.24	39.42
	PXN	Missense	c.1736G>A	p.Arg579Lys	54.91	59.68
	PTPRZ1	Missense	c.4492A>G	p.Met1498Val	54.55	49.14
	PLXND1	Missense	c.677G>T	p.Ser226Ile	54.47	57.80
	KIF12	LoF	c.1141delC	p.Gln381Serfs*52	54.21	29.27
	ELAC2	Missense	c.1009G>A	p.Val337Met	54.11	48.48
	USP16	Missense	c.1982A>G	p.Gln661Arg	53.85	53.64
	CACNA1D	Missense	c.6079G>A	p.Glu2027Lys	53.81	52.71

	GIT2	Missense	c.2122T>G	p.Tyr708Asp	53.78	50.34
	TSHZ2	Missense	c.2848G>A	p.Gly950Ser	53.69	42.94
	ITGA11	Missense	c.2141G>A	p.Arg714Gln	53.23	53.37
	ITPKA	Missense	c.434G>A	p.Ser145Asn	53.19	39.64
	TIMELESS	Missense	c.2722G>A	p.Asp908Asn	52.83	54.32
	LAMA1	Missense	c.4555G>C	p.Gly1519Arg	52.73	54.55
	USF3	Missense	c.6055G>A	p.Gly2019Ser	52.63	44.44
	PCNX3	Missense	c.4705G>A	p.Val1569Met	52.52	52.53
	ZNF827	Missense	c.2539T>G	p.Cys847Gly	50.98	41.86
	SEC16A	Missense	c.2717A>G	p.Asn906Ser	50.48	54.46
	SHROOM3	Missense	c.1609G>A	p.Asp537Asn	50.46	58.97
	MAG	Missense	c.1025C>T	p.Thr342Met	49.77	48.04
	NOS1	Missense	c.2066C>A	p.Ser689Tyr	49.39	49.42
	ANKRD44	Missense	c.2869G>A	p.Val957Ile	48.82	58.06
	KRT72	Missense	c.1433C>A	p.Ala478Glu	48.78	47.14
	GPR137	Missense	c.749C>T	p.Thr250Ile	48.56	43.84
	UBOX5	Missense	c.1357C>G	p.Gln453Glu.	48.18	42.71
	FAM83G	Missense	c.634G>A	p.Val212Ile.	47.78	43.04
	GCN1	Missense	c.5269A>G	p.Ile1757Val	46.56	40.30
	ALPK2	LoF	c.2111T>G	p.Leu704Ter	46.35	36.17
	NDUFS3	Missense	c.79C>T	p.Pro27Ser	46.20	39.04
	MROH8	Missense	c.451C>G	p.Gln151Glu	45.98	55.68
	LILRB1	Missense	c.1633G>C	p.Gly545Arg	45.90	34.03
	PLCB1	Missense	c.3619A>G	p.Ile1207Val	44.44	52.81
	MINAR1	Missense	c.1492T>A	p.Tyr498Asn	43.75	36.09
	KMT5C	Missense	c.1373G>A	p.Gly458Asp	43.09	37.14
	KRT85	Missense	c.220G>A	p.Gly74Ser	42.70	44.09

	ENTREP1	Missense	c.1222C>T	p.His408Tyr	41.46	41.74
	BAG1	Missense	c.340G>A	p.Glu114Lys	41.04	43.66
	NUDT8	Missense	c.633G>C	p.Glu211Asp	41.04	44.62
	TASOR	Missense	c.3527A>C	p.His1176Pro	38.54	49.41
	IKZF1	LoF	c.234A>T	p.*78Cysext*67	37.61	26.73
	PDIA5	Missense	c.302T>G	p.Leu101Arg	37.50	56.98
	UBXN6	Missense	c.1033G>A	p.Asp345Asn	33.79	35.71
	VPS26C	Missense	c.707A>G	p.Gln236Arg	32.10	49.02
	SMARCA4	Missense	c.4433C>T	p.Pro1478Leu	31.73	36.67
	ODF3L2	Missense	c.127G>A	p.Gly43Ser	31.58	38.46
	DTX3	Missense	c.73G>C	p.Val25Leu	31.16	41.67
	TBX21	Missense	c.1241G>C	p.Gly414Ala	30.70	30.65
	DYNC1H1	Missense	c.4964A>C	p.Lys1655Thr	28.81	40.22
	H2AC21	Missense	c.260C>T	p.Ala87Val	22.67	28.02
	TSC2	LoF	c.3490G>T	p.Ala1164Ser	21.95	6.12
	TULP3	Missense	c.232C>T	p.His78Tyr	21.64	32.61
	SLC22A18	Missense	c.946A>G	p.Ile316Val	20.99	43.33
	SARS2	Missense	c.127C>T	p.Leu43Phe	17.13	7.86
	NCOA1	LoF	c.3814C>T	p.Pro1272Ser	16.67	19.04
	MAP7	Missense	c.2186G>C	p.Gly729Ala	16.50	5.29
	SCN9A	Missense	c.4994A>G	p.Asn1665Ser	16.20	36.53
	WSCD1	Missense	c.482C>T	p.Ala161Val	14.81	81.61
	CYP4B1	Missense	c.425C>T	p.Thr142Ile	14.29	6.62
	OPRL1	Missense	c.922G>A	p.Gly308Ser	13.68	9.30
	FGFR4	Missense	c.760G>A	p.Gly254Arg	13.63	23.52
	PIGS	Missense	c.181C>A	p.Leu61Ile	13.17	17.77
	TEX37	Missense	c.148G>A	p.Asp50Asn	12.73	6.59

	PRDM13	Missense	c.1505C>T	p.Pro502Leu	11.26	17.33
	OPRM1	Missense	c.1321_1338del	p.Gly441_Ser446del	10.32	3.53
	USP17L22	Missense	c.875C>A	p.Ala292Asp	3.61	3.31
Patient exclusive alterations	*TAGAP*	Missense	c.1924C>T	p.His642Tyr	93.90	-
	KIF18A	Missense	c.1487A>G	p.Lys496Arg	80.85	-
	MPI	Missense	c.1000T>C	p.Tyr334His	56.15	-
	BPHL	Missense	c.394_396delAAG	p.Lys132del	54.29	-
	PDCL	Missense	c.678T>G	p.Ser226Arg	52.83	-
	EDRF1	Missense	c.1276G>A	p.Ala426Thr	51.08	-
	PTPRQ	Missense	c.5565T>G	p.Phe1855Leu	49.44	-
	PCM1	Missense	c.5666A>G	p.Lys1889Arg	45.00	-
	DOK2	Missense	c.235C>T	p.Arg79Trp	41.94	-
	MUC16	Missense	c.10513G>A	p.Val3505Met	37.90	-
	KMT2C	Missense	c.4384G>T	p.Gly1462Cys	37.72	-
	KRT10	Missense	c.1683_1684ins(90)	p.Gly565_His566ins30	25.72	-
	RECQL4	Missense	c.1592C>A	p.Ser531Tyr	16.00	-
	CRB1	Missense	c.1147T>G	p.Cys383Gly	9.38	-
	OR3A1	Missense	c.274A>G	p.Lys92Glu.	9.06	-
	MUC12	Missense	c.13094C>G	p.Thr4365Arg	8.78	-
	DDIT3	Missense	c.368A>C	p.Lys123Thr	8.57	-
	CFAP54	Missense	c.5867A>C	p.His1956Pro	8.33	-
	GOLGA6L2	Missense	c.1508_1509ins(18)	p.Glu502_Gln503ins6	8.14	-
	COG1	Missense	c.2623C>G	p.Leu875Val	7.27	-
	MUC6	Missense	c.4472A>C	p.Lys1491Thr	7.14	-
	BMP1	Missense	c.1843A>C	p.Lys615Gln	6.93	-
	BCL2L10	Missense	c.398A>C	p.Asp133Ala	6.59	-
	MUC16	Missense	c.40620G>C	p.Lys13540Asn	6.58	-

	WDR53	Missense	c.202T>G	p.Tyr68Asp	6.38	-
	PRB4	Missense	c.158C>A	p.Pro53His	5.08	-
	STEAP1	Missense	c.536G>C	p.Ser179Thr	5.06	-
	MUC12	Missense	c.9845C>G	p.Thr3282Arg	3.57	-
	RP1L1	Missense	c.4154T>A	p.Leu1385Gln	1.70	-
	KCNJ12	Missense	c.816T>G	p.His272Gln	1.14	-
PDX exclusive alterations	*RFPL1*	Missense	c.466C>T	p.Arg156Trp.	-	29.23
	ZNF618	Missense	c.917C>G	p.Pro306Arg	-	19.77
	NOS1AP	Missense	c.516C>A	p.Ser172Arg	-	14.29
	SFMBT2	LoF	c.1809-1G>T	n.a.	-	12.05
	PPP1CC	Missense	c.680T>C	p.Phe227Ser	-	9.60
	OPRK1	Missense	c.1060C>T	p.Arg354Trp	-	7.62
	RAG2	Missense	c.460G>T	p.Val154Phe	-	6.90
	KCNH2	Missense	c.2023A>T	p.Thr675Ser	-	5.36
	LAMB1	Missense	c.2326G>T	p.Asp776Tyr	-	2.53

LoF = Loss of function; n.a. = not available
